# An experimental investigation of social risk preferences for health

**DOI:** 10.1007/s11238-023-09928-w

**Published:** 2023-04-27

**Authors:** Arthur E. Attema, Olivier L’Haridon, Gijs van de Kuilen

**Affiliations:** 1 Erasmus School of Health Policy and Management (ESHPM), EsCHER, Erasmus University, P.O. Box 1738, 3000 DR Rotterdam, The Netherlands; 2 Univ Rennes, CNRS, CREM -UMR 6211, France and Institut Universitaire de France, F-35000 Rennes, France; 3 Tilburg School of Economics and Management, Tilburg University, Tilburg, The Netherlands

**Keywords:** Social risk, Ex-ante social welfare, Ex-post social welfare, Risk apportionment

## Abstract

In this paper, we use the risk apportionment technique of Eeckhoudt, Rey and Schlesinger (2007) to study higher order risk preferences for others’ health as well as ex-ante and ex-post inequality preferences for social risky distributions, and their interaction. In an experiment on a sample of university students acting as impartial spectators, we observe risk aversion towards social health losses and a dislike of ex-ante inequality. In addition, evidence for ex-post inequality seeking is much weaker than evidence for ex-ante inequality aversion. Because ex-ante inequality aversion is unrelated to risk aversion, we conclude that simple forms of utilitarianism are not relevant for individual judgment of social risk over health. Last, our investigation of precautionary distribution, which would occur when one particular group in the society suffers from background health risk, shows substantial polarization of preferences.

**Supplementary Information:**

The online version contains supplementary material available at 10.1007/s11238-023-09928-w.

## Introduction

In the health domain, the relations between preferences toward risk and inequality, i.e., individual preferences toward social risk, are still largely unexplored. Moreover, the few existing studies mostly relate risk aversion and inequality preferences for health (Abásolo & Tsuchiya, 2020; Attema et al., 2023; Keller & Sarin, 1988), but do not consider higher order risk attitudes such as prudence or temperance. However, knowledge of the whole spectrum of risk attitudes in a social context is crucial in the theoretical approaches to social welfare and redistributive policies in the presence of background risk (Courbage & Rey, 2012; Trautmann and van de Kuilen, 2022). For example, when some members of a society face a background risk, higher order risk preferences for social risk matter to justify an extra redistribution towards those members for precautionary purposes. In addition, as Konow (2003) pointed out, knowledge of the actual values and preferences of the members of a society is essential to any normative theory that claims to be relevant for allocative purposes, and so does the interaction between higher order risk preferences toward social risk and attitudes toward redistribution.

In this paper, we report on the measurement of (higher order) social risk preferences and various forms of inequality attitudes over life year distributions. We pay special attention to the difference between ex-ante inequalities, i.e., inequalities existing before the resolution of uncertainty, and ex-post inequalities, i.e., inequalities in realized outcomes after the resolution of uncertainty. In an experiment, we use the risk apportionment method introduced by Eeckhoudt and Schlesinger (2006) and Eeckhoudt et al. (2007) to elicit risk attitudes and attitudes towards inequality, alongside redistributive behavior when some members of the society face background risk. This allows us to test the theoretically predicted relationship between social risk preferences and preferences for redistribution in the health domain. The experiment implements an impartial spectator design, in which participants make decisions to allocate risks and health outcomes among two population groups. The design is meant to capture the preferences over allocations of an impartial spectator, with no direct stakes in the allocation to rule out potential confounds in our measurement of social risk preferences due to selfish motives.

The results of our experiment show substantial social risk aversion for health outcomes. We also find a significant association between social risk aversion and ex-post inequality attitudes and between risk aversion and prudence. Participants expressed a substantial aversion to ex-ante inequality. Still, this trait was not significantly related to risk preferences, casting doubt on the relevance of simple utilitarian social welfare functions to represent social risk preferences for health. Last, redistributive behavior was found to be driven mainly by the differences in longevity between individuals, and the mere presence of uncertainty did not, in the aggregate, universally trigger more redistributive behavior toward the person facing the background risk.

The remainder of this article is structured as follows. Section 2 summarizes related literature on this topic, followed by a description of the experimental design in Sect. 3. The results are presented in Sect. 4. Section 5 provides a discussion, and Sect. 6 concludes.

## Related literature

In the theoretical literature, attitudes toward social risk have been primarily investigated under the lens of expected utility. The seminal work by Harsanyi (1955) provides a simple representation of social welfare as a linear, additively separable combination of individual utilities. In this setting, attitudes towards inequality are directly linked to attitudes toward risk, e.g., diminishing (increasing) marginal utility implies a preference for an equal (unequal) distribution of wealth across members of society.

While relatively simple, this purely utilitarian approach has been criticized on many grounds. First, the utilitarian representation does not generally satisfy the Pigou-Dalton principle of transfers, which is a crucial element for modeling redistribution towards the worse-off individuals (Adler, 2011). Second, central to the present paper, with equal weights put on individuals, the utilitarian social welfare function does not distinguish between risk attitudes and attitudes towards inequality. Indeed, the utilitarian approach does not allow a distinction between ex-ante and ex-post inequality attitudes (Diamond, 1967; Fleurbaey, 2010; Keller & Sarin, 1988). In the ex-ante approach, the relevant inequality lies in the expected utilities across individuals.

In contrast, in the ex-post approach, the relevant inequalities refer to the actual utilities across individuals within the different states of the world. The utilitarian social welfare function predicts indifference between these two approaches of inequalities under risk.^1^ Over and above theoretical critics, empirical evidence provides mixed evidence, at least for monetary outcomes, on the relevance of a utilitarian criterion. For example, Amiel and Cowell (1992) find weak support for the Pigou-Dalton principle of transfer (around 35%), when it is tested as a preference for a mean-preserving spread of social outcomes, but a greater consensus (around 60%) when participants are asked to express a judgement on the theoretical definition of the principle of transfer. Consistent with the latter observation, experimental evidence provided by Traub et al. (2009) provides a rejection rate for at least 60% of the subjects for the utilitarian criterion. Cettolin and Riedl (2016) find a similar rejection rate of 64% for a parametric version of utilitarianism. In a seminal work on social risk preferences, Bernasconi (2002) questions Harsanyi’s theory of impartial preferences and expected utility theory using the same design and different frames. Bernasconi (2002) uses a questionnaire with nine binary choice tasks among hypothetical distributions. In one treatment, distributions are framed as social allocations of income to be chosen by an impartial observer. In another treatment, identical distributions are framed as lotteries on individual outcomes. Bernasconi (2002) finds that, even if strongly connected, social attitudes toward inequality cannot be only explained by individual attitudes toward risk. This structural difference between risk attitudes and perception of inequalities was also found by Bosmans and Schokkaert (2004). Bernasconi (2002) and Bosmans and Schokkaert (2004) also find that prioritarian alternatives to utilitarianism based on the principle of transfer do not offer a descriptive improvement either. In addition, both studies suggest a clear preference for randomization in social settings. These empirical results show that over and above pure risk preferences, attitudes towards inequalities involve a motive of procedural fairness and aversion to ex-ante inequality, advocated by, e.g., Diamond (1967), matter. This conclusion is also shared by Traub et al. (2009).

Most of the evidence regarding attitudes toward social risk has been collected in the domain of monetary outcomes; empirical evidence on social risk attitudes in the health domain remains scarce (Schokkaert and Tarroux, 2022). While it is well known that many individuals prefer an equitable distribution of health gains (Attema et al., 2015; Bleichrodt et al., 2004, 2005; Edlin et al., 2012), little remains known on the interaction between risk attitudes and attitudes towards health inequalities (Echazu & Nocetti, 2013; Johansson-Stenman et al., 2002). In a seminal paper, Keller and Sarin (1988) study the preference of a sample of university students towards the allocation of risks amongst 50 miners trapped in two separate mines. A majority of subjects chose to attempt to rescue the miners in both locations with a 50–50 percent chance of losing all the miners rather than attempting to rescue, and save, 50 miners in one location chosen at random. Most subjects preferred the first “common fate” option, which is riskier but less unequal from an ex-post perspective. In other words, subjects have a stronger aversion to ex-post inequality than to social risk.^2^ Abásolo and Tsuchiya (2020) use face-to-face interviews of a representative sample of the Spanish population to study ex-post inequality aversion and social risk aversion for health and wealth. They found significant differences between domains as well as differences between ex-post inequality aversion and social risk aversion. In particular, contrary to Keller and Sarin (1988), Abásolo and Tsuchiya (2020) find that aversion to ex-post inequality is weaker than aversion to social risk in the health domain.^3^

Finally, Trautmann and van de Kuilen (2022) study the distribution behavior of impartial spectators for monetary outcomes when one group of the population faces a background risk. They find that higher order risk attitudes have (weak) predictive power for behavior in the allocation task. Although they find support for a preference for equality, the presence of background risk does trigger precautionary redistribution in the domain of monetary gains. To the best of our knowledge, no previous study has investigated higher order social risk attitudes in the health domain.

## Method

### Risk apportionment

We assume preferences of the decision maker $\succcurlyeq$ depend on two attributes with non-negative quantities: the decision-maker is concerned with the longevity of two fully anonymous groups (group 1 and group 2) in the society. Preferences on the set of lotteries over longevities for both groups are assumed to be a weak order, and more longevity is always preferred to less. To characterize preferences over social risks, we base our analysis on the risk apportionment method introduced by Eeckhoudt and Schlesinger (2006) for univariate risk and Eeckhoudt et al. (2007) for multivariate risks. The risk apportionment method operationalizes (higher-order) risk preferences in terms of choices between two binary lotteries with equally likely outcomes that distribute harms and benefits differently. An example of an item revealing risk aversion for Group 1 is the following:

What is your most preferred alternative?

**Table:** 

Option A	Option B
50%: members of Group 1 live for 30 years members of Group 2 live for 40 years 50%: members of Group 1 live for 30 years members of Group 2 live for 40 years	50%: members of Group 1 live for 20 years members of Group 2 live for 40 years 50%: members of Group 1 live for 40 years members of Group 2 live for 40 years

In this example, Option A is riskless and Option B involves a mean-preserving spread of the same longevity. The longevity of members of Group 2, which is supposed to remain constant whatever the Option (A or B) and whatever the state of the world, is shown in grey. In terms of risk apportionment, starting from a baseline longevity of 40 years, Option A equally distributes a harm of 20 lost years over both states of the world (with 10 years lost with 50% chance in both states), while Option B assigns the whole harm to one state of the world and no harm to the other. It is worth noticing that both options not only involve individual risk for Group 1 but also, as a consequence, exhibit a different social distribution of outcomes. In Option A, ex-post inequality between groups is constant across states of the world, whereas in Option B, ex-post inequality is either large (20 years vs. 40 years) or null (40 years for both groups). In addition, both options have different levels of social risk, consistent with individual risk: in Option A, there is no social risk: total longevity is always equal to N*70 years. In Option B, there is social risk: total longevity is either equal to N*60 years or to N*80 years. Keeping longevity of members of Group 1 constant, risk aversion for Group 2 can be revealed with a similar item.

The risk apportionment method also allows for eliciting higher-order risk attitudes by adding different sources of uncertainty. For example, prudence for Group 2 can be elicited by the following choice:

What is your most preferred alternative?

**Table:** 

Option A	Option B
50%: members of Group 1 live for 40 years members of Group 2 live for 30 years 50%: members of Group 1 live for 40 years members of Group 2 live for 30 years OR 50 years	50%: members of Group 1 live for 40 years members of Group 2 live for 20 years OR 40 years 50%: members of Group 1 live for 40 years members of Group 2 live for 40 years

Starting from a baseline longevity of 40 years, the choice involves distributing a sure loss of 10 years and a zero-mean longevity risk of $\widetilde{t}=$±10 years over both states of the world (10 years lost for sure in one state, and the zero-mean risk $\widetilde{t}$ in the other state, Option A) or pooling both harms in one state (pooling together the sure loss and the zero-mean risk, Option B). In other words, the choice involves distributing $\widetilde{t}$ either to the high longevity state (Option A) or to the low longevity state (Option B). An individual choosing to disaggregate harms over the states of the world exhibits prudence for Group 2, while an individual choosing to aggregate harms over one state of the world exhibits imprudence for Group 2. As before, both options exhibit different social distributions of outcomes. Option A always exhibits ex-post inequality between groups, whereas in Option B, ex-post inequality is either large (20 years vs. 40 years) or null (40 years for both groups). In addition, both options have different characteristics of social risk: in Option A, total longevity is either equal to N*70 years with probability 3/4 or to N*90 years with probability 1/4, whereas in Option B, total longevity is either equal to N*60 years with probability 1/4 or to N*80 years with probability 3/4.

Eeckhoudt et al. (2007) show how the risk apportionment method can be extended to elicit (higher-order) cross-risk attitudes. An example of an item revealing correlation aversion for Group 1 and Group 2 is the following:

What is your most preferred alternative?

**Table:** 

Option A	Option B
50%: members of Group 1 live for 30 years members of Group 2 live for 40 years 50%: members of Group 1 live for 40 years members of Group 2 live for 30 years	50%: members of Group 1 live for 30 years members of Group 2 live for 30 years 50%: members of Group 1 live for 40 years members of Group 2 live for 40 years

This gamble involves risk for both members of Group 1 and Group 2 (30 or 40 years). The essential choice is if one prefers to combine the good outcome for Group 1 with the good outcome for Group 2, while at the same time combining the bad outcomes for both (Option B), or if one prefers to spread the risks and combine the good outcome for the one group with the bad outcome for the other group (Option A). The former is deemed *correlation seeking* and the latter *correlation aversion*. In a social setting, correlation averse and correlation seeking choices receive interpretations in terms of attitudes towards inequality and social risk. Indeed, in Option A, ex-post inequality is constant, and there is no social risk, whereas in Option B, there is no ex-post inequality but social risk.^4^

The structure of the risk apportionment method can be adapted to reveal more information on attitudes towards inequality. An example of an item revealing attitudes towards (ex-ante) inequality for Group 1 and Group 2 is the following:

What is your most preferred alternative?

**Table:** 

Option A	Option B
50%: members of Group 1 live for 30 years members of Group 2 live for 40 years 50%: members of Group 1 live for 40 years members of Group 2 live for 30 years	50%: members of Group 1 live for 40 years members of Group 2 live for 30 years 50%: members of Group 1 live for 40 years members of Group 2 live for 30 years

This choice involves risk for both members of Group 1 and Group 2 (30 or 40 years) in both options. Option B assigns the good outcome to Group 1 and the bad outcome to Group 2 whereas Option A distributes the risks among groups and assigns the good outcome to Group 1 in one state (i.e. 50% of the time) and to Group 2 in the other state (Option A). Both options share the same structure in terms of ex-post inequality (30 years vs. 40 years) and the same level of social risk (with total longevity equal to N*70 years whatever the option and the state of the world). The two options differ in terms of ex-ante inequality: from an ex-ante perspective, Option A offers the same prospect for members of Group 1 and members of Group 2 (30 years with 50% chance and 40 years otherwise). In contrast, Option B entails ex-ante inequality: the expected amount of longevity for Group 1 is 40 years but only 30 years for Group 2. As a consequence, choosing Option A reveals ex-ante inequality aversion and choosing Option B reveals ex-ante inequality seeking. The classical utilitarian framework à la Harsanyi assumes inequality aversion (ex-ante or ex-post) to be entirely driven by risk aversion. We tested this assumption with the association between attitudes towards ex-ante inequality, correlation aversion and risk aversion for both groups. If both attitudes towards inequality are similar and strongly associated with risk aversion, then a purely utilitarian approach is justified.

The risk apportionment technique can be extended to test cross-prudence and cross-temperance. For cross-prudence, one harm will be a sure loss and the other harm will be a zero-mean risk. For cross-temperance, both harms will be zero-mean risks. If the zero-mean risk on Group 2 is apportioned to the good outcome for Group 1, the individual reveals cross-prudence for Group 1, and, by extension, for social risk. If the zero-mean risk is apportioned to the bad outcome of the gamble for Group 1, the individual reveals cross-imprudence. Note that cross-prudence potentially generates substantial ex-post inequality compared to cross-imprudence since in the latter case, at least one state of the world exhibits no ex-post inequality. When the two harms are zero-mean risks, an individual who prefers to distribute zero-mean risks to each group over states of the world reveals cross-temperance. Here also, the cross-temperate choice is associated with a higher level of ex-post inequality.

We used the risk apportionment method to elicit the sign of the above-mentioned (higher-order) risk traits. Table 1 gives an overview of all traits we elicited. In Table 1, a couple ($$t-{t}_{1},t)$$ denotes the distribution of longevity between Groups 1 and 2 assigning $$t-{t}_{1}$$ to Group 1 and $$t$$ years to Group 2. In this table, Prospect 1 of the first row $$\left({t-{t}_{1},t)}_{0.5}(t-{t}_{2},t\right)$$ denotes a prospect where the distribution of longevity between groups 1 and 2 has 50% probability to be ($$t-{t}_{1},t)$$ and 50% to be ($$t-{t}_{2},t)$$. The other prospect of this first row is riskier for Group 1, since it involves a lower minimum ($$t-{t}_{1}-{t}_{2}$$) and a higher maximum ($$t$$). The other prospects can be interpreted similarly. For prudence, cross-prudence and cross-temperance $$\widetilde{t}$$, $$\widetilde{{t}_{1}}, \widetilde{{t}_{2}}$$, denote zero-mean risks on longevity.

**Table 1:** Overview of elicited traits

Trait if Prospect 1 is chosen	Prospect 1	Prospect 2
Risk aversion for Group 1^a,b,c^	$\left({t-{t}_{1},t)}_{0.5}(t-{t}_{2}t\right)$	$\left({t-{t}_{1}-{t}_{2},t)}_{0.5}(t,t\right)$
Risk aversion for Group 2^a,b,c^	$\left({t,t-{t}_{1})}_{0.5}(t,t-{t}_{2}\right)$	$\left({t,t-{t}_{1}-{t}_{2})}_{0.5}(t,t\right)$
Prudence for Group 2^a,d,e^	$\left(t,{t-{t}_{1})}_{0.5}(t,t+\widetilde{t}\right)$	$\left({t,t-{t}_{1}+\widetilde{t})}_{0.5}(t,t\right)$
Correlation aversion^a,b^	$\left({t-{t}_{1},t)}_{0.5}(t,t-{t}_{2}\right)$	$\left({t-{t}_{1},t-{t}_{2})}_{0.5}(t,t\right)$
Cross-prudence for Group 1^a,d^	$\left({t-{t}_{1},t)}_{0.5}(t,t+\widetilde{t}\right)$	$\left({t-{t}_{1},t+\widetilde{t})}_{0.5}(t,t\right)$
Ex-ante inequality aversion^a,b^	$\left({t-{t}_{1},t)}_{0.5}(t,t-{t}_{2}\right)$	$\left({t,t-{t}_{1})}_{0.5}(t,t-{t}_{2}\right)$
Cross-temperance^f^	$\left(t+\widetilde{{t}_{1}},{t)}_{0.5}(t,t+\widetilde{{t}_{2}}\right)$	$\left(t+\widetilde{{t}_{1}},{t+\widetilde{{t}_{2}})}_{0.5}(t,t\right)$

^a^$$t>{t}_{1}$$, ^b^$$t>{t}_{2}$$, ^c^ $$t>{t}_{1}+{t}_{2}$$,^d^$$E\left(\widetilde{t}\right)=0, t+\widetilde{t}>0$$, ^e^$$t+\widetilde{t}>{t}_{1}$$, ^f^$$E\left(\widetilde{{t}_{1}}\right)=0, t+\widetilde{{t}_{1}}>0$$,$$E\left(\widetilde{{t}_{2}}\right)=0, t+\widetilde{{t}_{2}}>0$$,$$t+\widetilde{{t}_{1}}+\widetilde{{t}_{2}}>0$$

### Social risk preferences and preferences for redistribution

Thus far, we have assumed fairly general preferences over longevity for both groups. To study distributional behavior, we add an additional assumption about preferences, namely that preferences satisfy bivariate expected utility theory. In what follows, let $u(x,y)$ denote the utility function defined over longevity for Group 1 ($x$) and Group 2 ($y$), and let ${u}_{x}(x,y)$ denote $\frac{\partial u\left(x,y\right)}{\partial x}$ and ${u}_{y}(x,y)$ denote $\frac{\partial u\left(x,y\right)}{\partial y}$. We follow the same subscript convention for the functions ${u}_{xx}(x,y)$, ${u}_{yy}(x,y)$, ${u}_{xy}(x,y)$, and so on.

In the context of expected utility theory, Eeckhoudt et al. (2007) and Jouini et al. (2013) show that the properties of the multi-attribute social utility function shape preferences for distributive justice. Eeckhoudt et al. (2007) show that the concavity of the utility function with respect to longevity of the two groups implies a preference for equalizing life expectancies, due to diminishing marginal utility. In a multivariate setting, knowledge of the sign of the second derivatives is not sufficient for equalization, because the marginal utilities also depend on attitudes towards correlation. Typically, correlation aversion (${u}_{xy}\left(x,y\right)<0$) implies that the marginal utility for the longevity of one of the groups decreases with increasing longevity for the other group. This additional impact of longevity on marginal utilities discourages the equalization of longevities for the two groups. Formally, equalization of longevity between the two groups requires the following condition to be verified:

1 $${u}_{xx}\left(x,y\right)+{u}_{yy}\left(x,y\right)<2{u}_{xy}\left(x,y\right).$$

In the experiment, we test this theoretical prediction by observing the decision maker’s redistribution behavior between two groups of unequal longevity and relate this behavior to the risk aversion and correlation aversion measures presented in Table 1. Starting from an unequal distribution of longevity $(t-{t}_{1},t-{t}_{2})$ with ${t}_{2}>{t}_{1}$, redistributive behavior can be elicited by the choice of $\tau$ such that members of Group 1 would live for $t-{t}_{1}-\tau$ and members of Group 2 would live for $t-{t}_{2}+\tau$. When condition $[1]$ is satisfied, the equalization transfer is equal to $({t}_{2}-{t}_{1})/2$.

Jouini et al. (2013) further demonstrate how adding a zero-mean risk to one of the groups can change the decision maker's redistributive behavior. Intuitively, when a zero-mean risk is added to one of the groups, precautionary distributional behavior emerges if the decision-maker is prudent. Indeed, a prudent decision-maker will be more likely to allocate more longevity to a beneficiary that faces a zero-mean risk, compared to the case where this risk is absent. However, as Jouini et al. (2013) show, the fact that preferences are multi-attribute requires that cross-prudence be taken into account as well. Cross-prudence implies a preference to handle a zero-mean risk for Group 2 when “richer” in terms of longevity for Group 1. Cross-prudence reduces the willingness to accept transfers between Group 1 and Group 2. Formally, the additional compensation for the group that deals with a zero-mean risk requires the following condition to be satisfied:

2 $${u}_{yyy}\left(x,y\right)<{u}_{xyy}\left(x,y\right).$$

In the experiment, we test this theoretical prediction by observing the decision maker's redistribution behavior between two groups of unequal longevity, one of which suffers from a zero-mean risk $\widetilde{t}$. Starting from an unequal distribution of longevity $(t-{t}_{1},t-{t}_{2}+\widetilde{t})$, with ${t}_{2}>{t}_{1}$, the decision-maker can redistribute a certain amount ${\tau }^{*}$ between the two groups. If condition $[2]$ is satisfied, the transfer with a zero-mean risk ${\tau }^{*}$ is higher than the transfer under certainty $\tau$. To test condition $[2]$, we relate the difference ${\tau }^{*}-\tau$ to the measures of prudence and cross-prudence presented in Table 1.

### Subjects and procedure

The sample consisted of students and alumni from Erasmus University Rotterdam. The first round of experiments was administered by 101 students from the Rotterdam Business School. This round, which took place just before the first COVID wave, was conducted in the laboratory, where four subjects at a time filled out the computerized survey under the supervision of an experimenter, who first read aloud the instructions. The second round of experiments was conducted with 45 students and recent graduates from healthy policy studies. This round had to be performed by means of individual online-interviews because of the COVID lockdown at the time of the second round of experiments. We used current and former health policy students as proxies for health policy makers, as many of these students end up working in this field, possible making decisions alike the ones used in the experiment. This allowed us to test if future health policy makers have similar preferences as students in a non-related field.

Subjects received instructions to complete a series of questions allocating reductions in life expectancy across two groups (denoted Group 1 and Group 2) of the same size, similar regarding age, gender, health status, etc. and completed four practice questions (one for risk aversion, one for correlation aversion, one for transfers under certainty and one for transfers under uncertainty). The order of the tasks was randomized. Within each trait, questions were not interspersed to avoid subjects having to switch between tasks continuously. Within each part, the questions were randomized. Five questions were repeated in order to test consistency (one question on risk aversion, one on prudence, one on correlation aversion, one on ex-ante inequality aversion and one on transfers under certainty). The experiment was programmed in Ztree. For in situ interviews, a researcher was in the room with the participants during all sessions, for online interviews, a researcher assisted the participants during a video call.

### Stimuli

For all tasks, we took a base longevity of *t* =  + 40 life years for both groups. Risk aversion for longevity for a particular group (e.g. Group 1) was assessed by implementing a mean-preserving spread in longevity between the options for that group while keeping longevity at *t* for the other group (e.g. Group 2). A similar procedure was used for the other traits. Table 2 shows the stimuli for all traits.

**Table 2:** Stimuli for the apportionment tasks

Taska	Trait	Prospect A	Prospect B
1*	Risk aversion for group 1	$\left( {40y - 10y, 40y} \right)_{0.5}$	${\left(40y-20y, 40y\right)}_{0.5}$
		$\left( {40y - 10y, 40y} \right)$	$(40y, 40y)$
2		$\left( {40y - 4y, 40y} \right)_{0.5}$	$\left( {40y - 8y, 40y} \right)_{0.5}$
		$\left( {40y - 4y, 40y} \right)$	$\left( {40y, 40y} \right)$
		${\left(40y-10y, 40y\right)}_{0.5}$	${\left(40y-14y, 40y\right)}_{0.5}$
3		$(40y-4y, 40y)$	$(40y, 40y)$
4	Risk aversion for group 2	${\left(40y, 40y-10y\right)}_{0.5}$	${\left(40y, 40y-20y\right)}_{0.5}$
		$(40y, 40y-10y)$	$(40y, 40y)$
5		${\left(40y, 40y-4y\right)}_{0.5}$	${\left(40y, 40y-8y\right)}_{0.5}$
		$(40y, 40y-4y)$	$(40y, 40y)$
		${\left(40y, 40y-10y\right)}_{0.5}$	${\left(40y, 40y-14y\right)}_{0.5}$
6		$(40y, 40y-4y)$	$(40y, 40y)$
7	Prudence for group 2	${\left(40y, 40y-10y\right)}_{0.5}$	${\left(40y, 40y-10y\pm 10y\right)}_{0.5}$
		$(40y, 40y\pm 10y)$	$(40y, 40y)$
8*		${\left(40y, 40y-10y\right)}_{0.5}$	${\left(40y, 40y-10y\pm 4y\right)}_{0.5}$
		$(40y, 40y\pm 4y)$	$(40y, 40y)$
9		${\left(40y, 40y-4y\right)}_{0.5}$	${\left(40y, 40y-4y\pm 10y\right)}_{0.5}$
		$(40y, 40y\pm 10y)$	$(40y, 40y)$
10*	Correlation aversion	${\left(40y-10\mathrm{y}, 40y\right)}_{0.5}$	${\left(40y-10\mathrm{y}, 40y-10y\right)}_{0.5}$
		$(40y,40y-10y)$	$(40y,40y)$
11		${\left(40y-10\mathrm{y}, 40y\right)}_{0.5}$	${\left(40y-10\mathrm{y}, 40y-4y\right)}_{0.5}$
		$(40y,40y-4y)$	$(40y,40y)$
12		${\left(40y-4\mathrm{y}, 40y\right)}_{0.5}$	${\left(40y-4\mathrm{y}, 40y-10y\right)}_{0.5}$
		$(40y,40y-10y)$	$(40y,40y)$
13*	Ex-ante inequality aversion	${\left(40y-10\mathrm{y}, 40y\right)}_{0.5}$	${\left(40y, 40y-10y\right)}_{0.5}$
		$(40y,40y-10y)$	$(40y,40y-10y)$
14		${\left(40y-10\mathrm{y}, 40y\right)}_{0.5}$	${\left(40y-10\mathrm{y}, 40y\right)}_{0.5}$
		$(40y,40y-10y)$	$(40y-10y,40y)$
15		${\left(40y-4\mathrm{y}, 40y\right)}_{0.5}$	${\left(40y, 40y-4y\right)}_{0.5}$
		$(40y,40y-4y)$	$(40y,40y-4y)$
13	Cross-Prudence for group 1	${\left(40y, 40y-10y\right)}_{0.5}$	${\left(40y\pm 10y, 40y-10y\right)}_{0.5}$
		$(40y\pm 10y, 40y)$	$\left(40y, 40y\right)$
14*		${\left(40y\mathrm{y}, 40y-10y\right)}_{0.5}$	${\left(40y\pm 4y, 40y-10y\right)}_{0.5}$
		$(40y\pm 4y, 40y)$	$\left(40y, 40y\right)$
15		${\left(40y, 40y-4y\right)}_{0.5}$	${\left(40y\pm 10y, 40y-40y\right)}_{0.5}$
		$(40y\pm 10y, 40y)$	$\left(40y, 40y\right)$
16	Cross-Temperance	${\left(40y\pm 10y, 40y\right)}_{0.5}$	${\left(40y\pm 10y, 40y\pm 10y\right)}_{0.5}$
		$(40y, 40y\pm 10y)$	$(40y, 40y)$
17		${\left(40y\pm 4y, 40y\right)}_{0.5}$	${\left(40y\pm 4y, 40y\pm 10y\right)}_{0.5}$
		$(40y, 40y\pm 4y)$	$(40y, 40y)$
18		${\left(40y\pm 10y, 40y\right)}_{0.5}$	${\left(40y\pm 10y, 40y\pm 4y\right)}_{0.5}$
		$(40y, 40y\pm 4y)$	$(40y, 40y)$

^a^choice tasks indicated by

*were repeated once as consistency checks

### Redistributive transfers

Transfer proneness between groups was operationalized as the preferred transfer level between Group 1 and Group 2. Here, participants were presented with an asymmetrical distribution of losses in longevity, with Group 2 suffering from a larger decrease in longevity than Group 1. At baseline (transfer $\tau$ of 0 years, i.e. no transfers), the asymmetry remained. Depending on the questions, longevity was either certain or associated with uncertainty. The former case represents the situation in which, e.g., genetic variation between the groups caused a known reduction in life duration for Group 2, whilst in the second case, the additional health risk was caused by a larger genetic variation. The subject could, however, influence the distributions of longevity by choosing a preferred redistributive transfer, represented as a number of expected life years ranging from 0 to 20, which subjects could choose from in steps of 1 year. We used three questions with a fixed duration, and three questions in which we added a zero-mean risk as a random duration $\widetilde{t}$ of either + 4 or − 4 years, equally likely. An overview of the stimuli is provided in Table 3. Screenshots of this task are shown in the Web Appendix. As can be seen from Table 3, Tasks 1 and 4 involve small losses, Tasks 2 and 5 involve large losses and Tasks 3 and 6 involve a large spread between the two groups. In what follows, we refer to those tasks as either small losses, larges losses or large spread, under certainty or uncertainty.

**Table 3:** Stimuli for the redistributive transfer

Difference in longevity	Tast^a^	Prospect in case of no transfer	Prospect in case of equalizing transfer^b^
	1	$(40y-4y, 40y-10y)$	$(40y-7y, 40y-7y)$
Certain	2	$(40y-10y, 40y-20y)$	$(40y-15y, 40y-5y)$
	3*	$(40y-4y, 40y-20y)$	$(40y-12y, 40y-12y)$
	4	$(40y-4y, 40y-10y\pm 4y)$	$(40y-7y, 40y-7y\pm 4y)$
Uncertain	5	$(40y-10y, 40y-20y\pm 4y)$	$(40y-15y, 40y-15y\pm 4y)$
	6	$(40y-4y, 40y-20y\pm 4y)$	$(40y-12y, 40y-12y\pm 4y)$

^a^choice tasks indicated by * were repeated once as consistency checks. ^b^ for Tasks 4 to 6 equalizing transfers are based on equalization of expected values

### Analysis

Data analysis was performed in R (R Core Team, 2016). We used the number of choices (out of 3) that are compatible with a given risk trait as our measurement of the strength of social risk preferences and inequality preferences. In our analysis, a subject was classified according to a social risk trait if the majority of her choices was consistent with that particular trait. For example, an individual was classified as being risk averse (seeking) for Group 1 if most of her choices were compatible with risk aversion (seeking). For each of these traits, we investigated whether people showed a given risk preference or behaved at random based on a chi-square test. At the aggregate level, we report the average percentage of choices over tasks compatible with each trait. We use chi-square tests to compare the classifications obtained for each trait and Student t tests to assess the differences between health policy students and other students. We estimated a logit model on risk apportionment as a complementary analysis.

We describe the redistributive behavior of subjects in four ways. First, we used a classification of individuals based on the comparison of their preferred redistributive transfer τ (under certainty) or ${\tau }^{*}$ (in the presence of a zero-mean risk) with the equalizing transfer $({t}_{2}-{t}_{1})/2$.^5^ Second, we computed the difference between transfers ${\tau }^{*}-\tau$, and classified individuals as having a constant (increasing, decreasing) profile if they reported the same (increasing, decreasing) transfer for the tasks under risk and under certainty. For both classifications, we used a strict rule requiring three out of three comparisons to fulfil each condition. We also used a more lenient rule that is satisfied if at least two out of three comparisons fulfil the conditions. Third, we tested conditions [1] and [2] based on the difference between transfers ${\tau }^{*}-\tau$ and the answers to the risk apportionment tasks. We assumed that condition [1] was fulfilled if the number of univariate risk-averse choices for both groups was smaller than twice de number of correlation averse choices. Similarly, we assumed that condition [2] was fulfilled if the number of univariate prudent choices for group 2 was smaller than the number of cross-prudent choices (for group 2). Last, we linked redistributive transfers τ and ${\tau }^{*}$, and the difference ${\tau }^{*}-\tau$, with the classification of individuals based on their risk apportionment choices with a mixed-effects linear model. Fixed factors were student status (health policy student vs. non-health policy student), risk attitudes (risk aversion, correlation aversion, prudence), and context (risk vs. certainty).^6^ Participants were treated as a random effect.

## Results

### Consistency

To assess whether participants were consistent in their answers, five items were included twice in the experiment measuring risk aversion, prudence, correlation aversion, ex-ante inequality and transfer policy. For binary choices, subjects made the same choice in 80.48 percent of the repeated choices, a rate in line with the consistency rates observed in similar experiments. Some variability existed between the different tasks: consistency was higher for ex-ante inequality (88.36 percent) and lower for prudence (75.34 percent). For transfer policy choices, participants made the same choice in 76.71 percent of the repeated choices. Allowing for an error margin of 5 percentage points, the consistency rate increases to 88.36 percent. Individual-level analysis of consistency rates shows systematic differences in consistency rates between health policy students and students in the control group (84.44 percent vs. 77.62 percent, resp., *t*-test *p* = 0.03).

### Risk preferences and attitudes towards inequality

Table 4 shows the results on risk preferences and attitudes towards ex-ante inequality. The first two columns show the aggregate results: the mean proportion of the three choices compatible with each trait and the associated standard deviation. The last two columns show the individual results. The third column corresponds to the classification of individuals, based on their risk preferences, and the fourth shows the p-value of a one-sided binomial test for comparison between the percentage of individuals and 50 percent. At the aggregate level, we performed a series of chi-squared tests to check whether the observed distribution of preferences deviated from the distribution that would be observed if subjects chose randomly. All tests show that choices were not made at random.

**Table 4:** Risk preferences and attitudes towards inequality: aggregate results and individual classification

Trait	Aggregate results		Individual classification	
	Mean	sd	Proportion	*p*-value
Risk aversion for group 1	65.98	6.65	69.86	< 0.01
Risk aversion for group 2	63.70	1.81	61.64	0.00
Prudence	55.71	2.77	56.16	0.08
Correlation aversion	52.74	2.47	52.05	0.34
Ex-ante inequality aversion	81.96	0.79	84.25	< 0.01
Cross-prudence	57.31	2.20	58.90	0.02
Cross-temperance	45.21	3.14	45.89	0.18

We found social risk aversion to be the predominant pattern for both group 1 and group 2, with around a two-third majority of the choices compatible with risk aversion in both cases. Although health policy students displayed a slightly higher degree of risk aversion for both groups, the difference between health policy students and students in the control group was not significantly different from zero. We found a clear association between risk attitudes for group 1 and risk attitudes for group 2 (Chi-square test, *p* < 0.01), which supports anonymity with respect to risk aversion. For the remainder, unless stated otherwise, we pooled the choices in both risk aversion tasks.^7^

The evidence for prudence for longevity was much weaker. Indeed, only a small, non-significant, majority of participants were classified as prudent (for group 2). This feature was shared by both health policy students and students in the control group. Results show an association between risk aversion and prudence for longevity (Chi-square test, *p* = 0.02).^8^

**Fig. 1:**
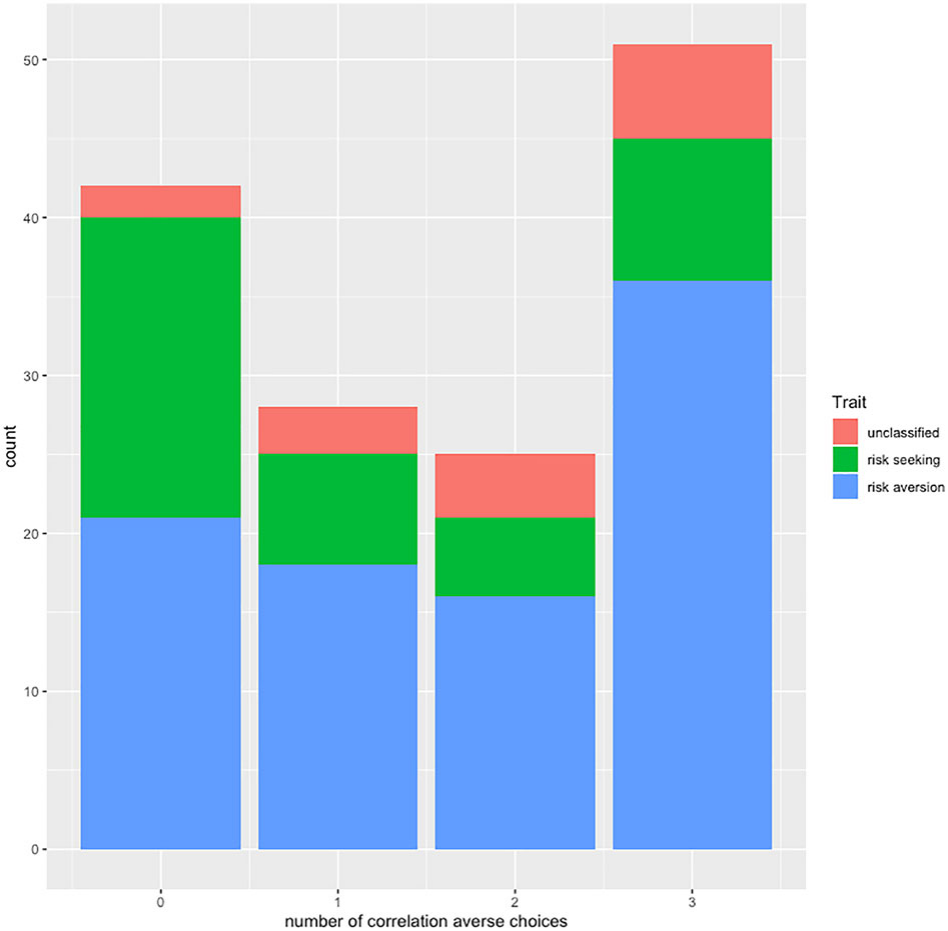
Correlation aversion and risk attitudes: distribution of the number of correlation averse choices

We found no clear choice pattern indicative of a preference for correlation aversion for longevity between the two groups with only 52.74 percent of the choices compatible with correlation aversion. Figure 1 shows the distribution of correlation averse choices for groups 1 and 2 together with the distribution of risk-averse individuals. It displays two notable features. First, correlation aversion, which corresponds to ex-post inequality seeking, is associated with risk aversion (Chi-square test, *p* = 0.03). The association was much stronger for health policy students than for students in the control group (Chi-square test, *p* = 0.02 vs. *p* = 0.42). Second, the figure shows a clear polarization of preferences between correlation averse (i.e., ex-post inequality seeking) and correlation seeking (ex-post inequality averse) individuals.

We found no evidence for cross-prudence to be a majority trait. We observed a significant association between cross-prudence, prudence (Chi-square test, *p* = 0.01) and correlation aversion (Chi-square test, *p* < 0.01), while the association between cross-prudence and risk aversion was only marginally significant. Evidence was similar for cross-(in)temperance: a small, non-significant, majority of subjects made choices compatible with cross-intemperance, a trait associated with cross-imprudence (Chi-square test, *p* < 0.01) and correlation seeking (Chi-square test, *p* < 0.01). The combinations of either correlation aversion, cross-prudence and cross-temperance or correlation seeking, cross-imprudence and cross-intemperance captured the majority of multivariate risk preferences (for 30.14 percent and 26.03 percent of subjects, respectively).

Last, we found strong evidence in favor of aversion to ex-ante inequality for longevity both at the aggregate and at the individual level: most individuals were classified as averse to ex-ante inequality (84.25 percent of individuals). We found no significant association between attitudes towards inequality and risk preferences (i.e., risk aversion, prudence, correlation aversion, cross-prudence and cross-temperance).

In order to identify a systematic difference between health policy students and students from the control group, we estimated a multilevel logit regression model with risk and inequality apportionment as the dependent variable, student status (health policy student vs. non-health policy student) and trait as fixed factors, subject and task being treated as random effects. Table A1 in the Appendix shows the results of the estimation. Health policy students opted more frequently for apportionment than students in the control group, but the effect was only marginally significant.

### Redistributive transfers with certain differences in longevity

**Fig. 2:**
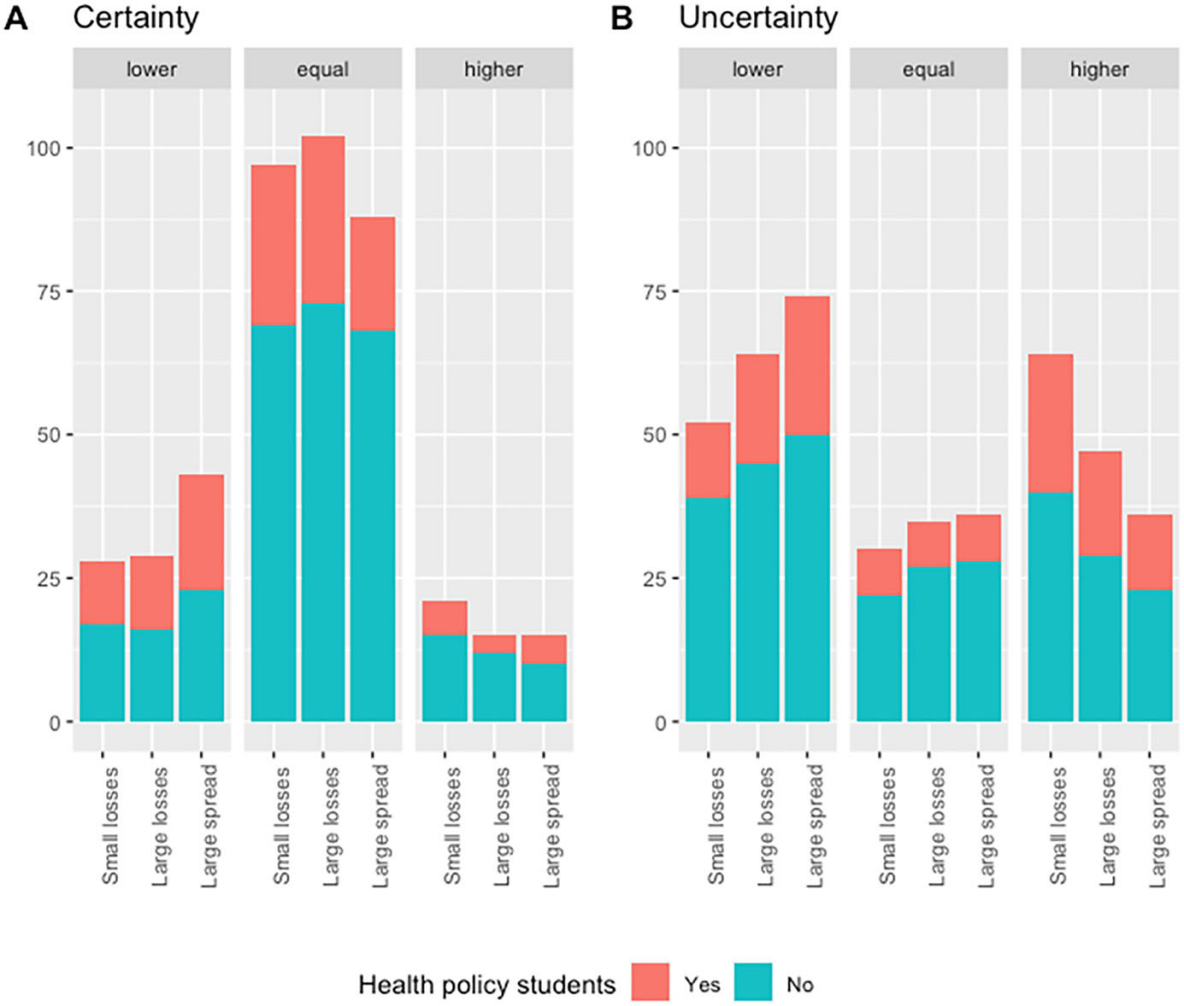
Distributions of transfers between health-policy tasks under certainty (panel A) and uncertainty (panel B) Note: distributions of transfers between groups under certainty (panel A) and uncertainty (panel B). Each panel shows the distribution of transfers lower than, equal to and higher than the (expected-value) equalizing transfer for each task. The small losses task corresponds to − 4 vs. − 10 years of lost longevity. The large losses task corresponds to − 10 vs. − 20 years of lost longevity. The large spread task corresponds to − 4 vs. − 20 years of lost longevity

Table 6 shows the descriptive statistics on the choice of health policy transfers and Fig. 2, panel (A) shows the number of subjects who chose transfers lower, equal or higher than the amount necessary to equalize longevity between groups. Table 6 shows that the median transfer corresponded to the transfer needed to equalize longevity between groups: three years in the small losses task (− 4 vs. −10), five years in the large losses task (− 10 vs. − 20) and eight years in the large spread task (− 4 vs. − 20). Table 6 also shows the results of a Student t-test on the equality between the chosen level of transfers and the equalizing transfer value. We found transfers to be significantly lower in the large spread task. In that case, the amount transferred was significantly lower than the equalizing value. Between-subject comparisons between health policy students and students in the control group show no significant difference in the chosen level of transfers.

**Table 6:** Descriptive statistics on the choice of transfer intensity

Differences in longevity		Certain			Uncertain	
	Small losses	Large losses	Large spread	Small losses	Large losses	Large spread
Median	3.00	5.00	8.00	3.00	5.0	7.00
Mean	3.02	4.92	7.45	3.48	4.9	7.04
Standard deviation	1.55	2.27	2.51	2.10	2.2	3.11
Equality with equalizing value (p-value)	0.87	0.66	0.01	0.01	0.6	0.00

The small losses task corresponds to − 4 vs. − 10 years of lost longevity. The large losses task corresponds to − 10 vs. − 20 years of lost longevity. The large spread task corresponds to − 4 vs. − 20 years of lost longevity. The equalizing value is defined as the expected value equalizing transfer

### Redistributive transfers with uncertain differences in longevity

The comparison of Panels (A) and (B) in Fig. 2 shows that introducing uncertainty with a zero-mean risk generates a sharp decrease in the number of equalizing transfers (in expected value) between group 1 and group 2. Introducing uncertainty increased both the number of transfers lower and higher than the equalizing transfer. For the small losses task, introducing a zero-mean risk for group 2 increased the amount of the transfer (Student *t*-test, *p*-value = 0.01). On the opposite, for the large losses task, we found no significant difference in transfers between uncertainty and certainty (Student *t*-test, *p*-value = 0.95). Last, introducing a zero-mean risk for group 2 in the large spread task decreased the amount of the transfer between group 1 and group 2 (Student *t*-test, *p*-value = 0.04). A classification of individuals based on their transfer between certainty and uncertainty confirms the large decrease in equalizing transfers. Individual analysis from Table 7 shows that only a minority of individuals kept the same transfer between certainty and uncertainty. Even with the more lenient classification rule, individuals choosing constant transfers remain a minority. Still, no systematic behavioral pattern emerged between decreased transfers, equal transfers and increased transfers.

**Table 7:** Classification of individuals based on their transfers between certainty and uncertainty

	Decreased transfers	Equal transfers	Increased transfers	Mixed
Strict rule	16	22	13	95
Lenient rule	49	37	43	17

Table 6 shows that the standard deviation of the transfer increases when a zero-mean risk was introduced in the small losses and large spread tasks. We tested this increase with a Pitman-Morgan test of variances for paired samples and found the increase in the variance of transfers to be significant for both tasks (*p*-value = 0 for both).^9^

In order to test the predictions of conditions [1] and [2], we measured the association between transfers and the classification of individuals based on their choice apportionment in binary choice with a mixed-effect linear model. We estimated the model for each task (small losses, large losses and large spread) separately and for the three tasks pooled together. Table 8 presents the results of the estimations. It shows that behavioral traits fail to reach significance, risk for group 2 being the main significant effect on transfers. For instance, risk aversion for group 2 consistently increased the amount of transfer when group 2 suffered an additional risk, but this effect was far from reaching significance. Controlling for behavioral traits in the model estimated on all tasks shows that relative to the small losses tasks, both large losses and large spread tasks involve smaller transfers from group 1 to group 2, an effect consistent with Fig. 2.

**Table 8:** Transfers between groups and behavioral traits, estimation results

	Small loss	Large loss	Large Spread	All
(Intercept)	0.13	− 0.40	− 1.33^*^	− 0.09
	(0.40)	(0.46)	(0.65)	(0.44)
Health policy student	0.23	− 0.12	− 0.10	0.01
	(0.29)	(0.32)	(0.47)	(0.31)
Risk for group 2	0.46^**^	− 0.01	− 0.40^*^	0.01
	(0.16)	(0.23)	(0.20)	(0.12)
Risk aversion	0.14	0.26	0.41	0.27
	(0.27)	(0.31)	(0.45)	(0.29)
Prudence	0.05	0.34	− 0.09	0.10
	(0.28)	(0.31)	(0.45)	(0.30)
Correlation aversion	0.36	0.35	− 0.07	0.21
	(0.28)	(0.32)	(0.47)	(0.30)
Cross-prudence	− 0.12	− 0.56	− 0.26	− 0.31
	(0.29)	(0.33)	(0.49)	(0.32)
Inequality aversion	− 0.49	0.18	0.94	0.21
	(0.36)	(0.41)	(0.60)	(0.39)
Large loss task				− 0.34^*^
				(0.15)
Large spread task				− 1.01^***^
				(0.15)
AIC	1176.78	1309.32	1370.72	3782.22
BIC	1213.55	1346.09	1407.48	3839.52
Log Likelihood	− 578.39	− 644.66	− 675.36	− 1879.11
Num. obs	292	292	292	876
Num. groups: subject_number	146	146	146	146
Var: subject_number (Intercept)	1.50	1.20	5.30	2.34
Var: Residual	1.93	3.83	2.80	3.23

^***^p < 0.001; ^**^p < 0.01; ^*^p < 0.05

## Discussion

In this study, we have shown how higher order risk preferences toward social risk and attitudes toward redistribution interact. We have measured higher order risk preferences with the risk apportionment method and measured willingness to implement Pigou-Dalton transfers for health in different risky and riskless contexts.

*Risk aversion for social health risks*. Regarding risk for each of the two groups used in our study, we found substantial social risk aversion. This finding complements -in a social setting- the well-established body of literature on individual risk aversion for health (Attema et al., 2013; Miyamoto & Eraker, 1985; van der Pol & Ruggeri, 2008; van Osch et al., 2004). In addition, we found evidence of prudence for social risk, but this trait was only marginally significant and less prominent than risk aversion. The experimental literature on individual risk attitudes for monetary gains offers several points of comparison to our findings. This literature tends to find more prudence than risk aversion (Deck & Schlesinger, 2014; Ebert & Wiesen, 2014; Noussair et al., 2014) but the opposite pattern has also been observed in several experiments (Krieger & Mayrhofer, 2012; Attema et al., 2019; Bleichrodt & van Bruggen, 2022; Ebert and van de Kuilen, 2015). In the loss domain, Bleichrodt and van Bruggen (2022) find less prudent than risk-averse choices and even significant imprudence in the aggregate. The latter result is also reported by Brunette and Jacob (2019). Last, the emerging literature on individual higher order risk preferences for health (Attema et al., 2019) finds a similar pattern to the one observed for monetary outcomes by Bleichrodt and van Bruggen (2022).

*Ex-ante vs. ex-post inequality aversion*. For risk attitudes across groups, we found strong evidence for ex-ante inequality aversion but almost no evidence for ex-post inequality aversion or inequality seeking. In the aggregate, the main trait was correlation aversion, which corresponds to ex-post inequality seeking. However, this trait was not strong enough to be significant. Rohde and Rohde (2015) find similar results in an experiment involving monetary gains. The majority preference of their sample of university students is consistent with ex-post inequality seeking, alongside ex-ante inequality aversion and risk aversion. In addition, in one of their choices comparing perfect positive correlation and perfect negative correlation, they find non-significant correlation seeking, a result identical to ours. Ebert and van de Kuilen (2015) use the risk apportionment method to investigate justice preferences over monetary outcomes in a similar setting to the one presented in this paper. Their modal choice pattern indicates a relatively strong preference for correlation seeking. We do replicate correlation seeking for health losses, but not with the strength found by Ebert and van de Kuilen (2015) for monetary gains. In addition, at the level of individual subjects, we found a clear polarization in favor of either ex-post inequality aversion or ex-post inequality seeking. In a somewhat different experimental design based on the allocation of lottery tickets between two impoverished Kenyan families, Andreoni et al. (2020) also find that the most common pattern of choice is to select the ex-ante fair alternative over the ex-post fair alternative. Furthermore, Andreoni et al. (2020) observe a strong framing effect: choices made before the resolution of uncertainty reveal ex-ante inequality aversion while choices made after some resolution of uncertainty reveal ex-post inequality aversion. Because uncertainty is never resolved in our experimental design, we cannot observe a similar framing effect.

*Inequality attitudes and social comparison*. Most experimental evidence of ex-ante and ex-post inequality aversion has been collected in settings where the decision-maker has stakes in the decision, and, in that case, social risk entails social comparison. In a probabilistic dictator game in which participants have the option to win a prize with a dummy player, Krawczyk and Le Lec (2010) find that dictators are less selfish when outcomes are independent than when they are perfectly negatively correlated. At the level of their subjects, neither aversion to ex-ante inequality nor to ex-post inequality can best explain the choices made by individuals. In an experiment designed to directly investigate how social comparison affects risk-taking, Bolton and Ockenfels (2010) find no evidence for ex-post inequality aversion and evidence for preference for ex-ante equality. Linde and Sonnemans (2015) report similar results. Closer to our experimental design, Ebert and van de Kuilen (2015) investigate fairness preferences in addition to justice preferences and other treatments. In their fairness treatment, the decision involves outcomes accruing to oneself and to another person. They found similar patterns of choice (risk aversion, prudence, and ex-post inequality seeking), irrespective of whether experimental subjects' monetary outcomes were involved in the decisions (the fairness treatment) or not (the justice treatment). Our experiment deals with health outcomes, and we used longevity as a proxy for these health outcomes. We chose not to implement a design with social comparison for a couple of reasons. First, personal health is a high-dimension outcome, and between-person comparisons are much more complex to perform than comparisons between monetary outcomes. Second, the latent heterogeneity in health conditions between participants in an experiment is potentially considerable and cannot be investigated for moral reasons by revealed-preference mechanisms. Last, social comparison experiments use real monetary incentives to generate an incentivized decision environment, which is not an option for health outcomes.

*Hypothetical stakes*. The fact that most of our findings with hypothetical incentives in the health confirm previous findings obtained under real incentives for monetary gains or losses suggests that the provision of monetary rewards does not appear to affect preferences toward higher-order social risk preferences. This observation has also been suggested by Trautmann and van de Kuilen (2018) for higher order individual risk. For hypothetical health outcomes, one key point for the internal validity of the measures is the relevance of the scenario presented to participants in the experimental instructions. If subjects did not adhere to the instructions, they might answer randomly during the experiment. To check for randomness, we included several consistency checks in the experiment. We observed a relatively high consistency in those repeated tasks, suggesting that our experimental subjects did not answer randomly and adhered to the proposed scenario.

*Risk preferences and inequality attitudes*. Cross-comparison of risk attitudes and attitudes towards inequality also shows a different picture depending on whether ex-ante or ex-post inequality aversion is considered. We found no association between ex-ante inequality aversion and risk aversion and, more generally, no association with any other trait measured in our experiment. This finding confirms the previous literature on monetary outcomes (Bernasconi, 2002; Bosmans & Schokkaert, 2004) and casts doubt on the relevance of the utilitarian framework to study people’s fairness views of health inequalities. Our results are another proof, based on the risk apportionment elicitation technique, that one should be careful when inferring inequality attitudes from risk attitudes, even when measured in a social setting. Conversely, we found a clear association between ex-post inequality seeking (correlation aversion) and risk aversion. Such a finding is not uncommon in the literature: Adam et al. (2014) find that when individual payoffs between the decision-maker and the other person are symmetrically coupled, subjects make more risky choices, whereas they make less risky choices when payoffs are asymmetrically coupled; Rohde and Rohde (2011) find that most of their risk-averse subjects prefer independent risk across members of the population rather than correlated risks. At odds with our results, Koch et al. (2021) find that when two peers are endowed with identical binary lotteries and must choose whether to couple or uncouple these lotteries, correlation seeking (i.e., coupling) is clearly associated with risk aversion. We also found evidence for cross-prudence but no significant evidence for cross-intemperance. Ebert and van de Kuilen (2015) found similar results for monetary outcomes. However, with the opposite significance: for monetary gains, they found non-significant evidence for cross-prudence and significant evidence for cross-intemperance. Given that our study and Ebert and van de Kuilen (2015) are the only studies measuring those traits by an impartial observer, the source of the difference in significance remains challenging to assess and calls for future research and replication.

*Pigou-Dalton transfers in the health domain*. In the second part of the experiment, we tested if subjects preferred precautionary redistribution by comparing the willingness to implement Pigou-Dalton transfers under certainty and uncertainty. While we found evidence for redistribution under certainty, except when the difference in initial health was substantial, the mere presence of uncertainty had ambiguous effects. A large minority of our experimental subjects transferred more to the group facing a zero-mean risk, while another, equivalent in size, minority of subjects transferred less in the presence of uncertainty. Under certainty, our results replicate Trautmann and van de Kuilen (2022)’s results for the monetary outcomes, with a strong tendency to equalize outcomes through Pigou-Dalton transfers. Under uncertainty, Trautmann and van de Kuilen (2022) find positive precautionary redistribution (i.e., more redistribution toward the person facing the background risk) for gains, but not for losses. Based on transfers with health losses, our results appear somehow intermediate, with a clear polarization of the respondents between a clear preference for precautionary redistribution and the absence of such preference.

*Model-free measures and expected utility*. One of the main purposes of the risk apportionment method of Eeckhoudt and Schlesinger (2006) and Eeckhoudt et al. (2007) is to offer a model-free procedure to elicit higher order risk preferences. The elicited traits can also be interpreted within an expected utility model, with a bivariate utility defined over longevity for each group. A typical example of an expected utility interpretation of our results would be the lack of evidence for a sign of the cross-derivative of the bivariate utility function revealed by the non-significant result on correlation seeking. Our measure of redistributive behavior can also be compared with the prediction of an expected utility model along the lines of Jouini et al. (2013). While our results do not contradict those predictions, the analysis exhibits a lack of significance that leads to a somewhat inconclusive result on the relevance of the expected utility model to study redistributive behavior and precautionary redistribution.

*Convenience sample and outbreak of COVID-19*. Because we implemented a novel method to study attitudes towards health inequalities under uncertainty, we used a convenience sample to test-bed the reliability and feasibility of the method. Our convenience sample regroups both health-policy students and other students. The outbreak of COVID-19, that occurred in the middle of our experiment, creates a possible confound in the experiment we cannot control for. Indeed, due to sanatory constraints, the health-policy students had to be interviewed in personal online interviews after the outbreak of the disease instead of lab sessions with a small-groups of students. Therefore, the distinctive behavior of the health-policy students, e.g., the evidence for more precautionary redistributive behavior, cannot be attributed to a fixed-effect of the outbreak of the disease, behavioral changes due to COVID-19 or the mere effect of health-policy studies.

## Conclusion

In this paper, we have shown how the risk apportionment technique can be applied to study (higher order) social risk preferences as well as ex-ante and ex-post inequality preferences for social risky distributions over life years. We implemented the technique to elicit risk aversion, correlation aversion and aversion to ex-post inequality, aversion to ex-ante inequality, (cross-) prudence and (cross-) temperance. We also use a Pigou-Dalton transfer approach to test for precautionary motives in redistribution. We observe that impartial spectators are risk averse towards social health losses and found large support for ex-ante inequality averse choices, but unrelated to risk aversion. Evidence for ex-post inequality seeking was much weaker. Our results show that simple forms of utilitarianism are not relevant for individual judgment over social risk over health, and more general expected utility models of redistribution might be better justified. Finally, individual results show a clear polarization of attitudes towards precautionary distribution, which is not, as a consequence, a universally supported social policy for health.

### Electronic supplementary material

Below is the link to the electronic supplementary material.

Supplementary file1 (PDF 508 kb)

## Data Availability

The data that support the findings of this study are available from the corresponding author upon request.

## Appendix

**Table A5:** Logit regression on risk apportionment

Intercept	− 0.36^*^
	(0.16)
Health policy student	0.40
	(0.22)
Risk aversion	1.00^**^
	(0.13)
Prudence	0.53^**^
	(0.15)
Correlation aversion	0.38^*^
	(0.15)
Cross-prudence	0.61^**^
	(0.15)
Aversion to ex-ante inequality	2.09^**^
	(0.17)
Log Likelihood	− 1796.95
Num. obs	3066
Num. groups: subject_number	146
Num. groups: trial	3
Var: subject_number (Intercept)	1.19
Var: trial (Intercept)	0.01

^**^*p* < 0.01; ^*^*p* < 0.05. Multilevel logit regression model on the probability to select the apportionment choice in a given task. Cross-temperance is the reference level.

## Footnotes

1 Suppose a social decision-maker has to choose between the two following alternatives taken from Keller and Sarin (1988): in Alternative 1, Person 1 dies and Person 2 lives; in Alternative B, there is a 50% chance that Person 1 dies and Person 2 lives and a 50% chance that Person 1 lives and Person 2 dies. Because the probability distribution over the number of fatalities is the same, expected utility predicts indifference between the two alternatives. Most persons, however, would have a strict preference for Alternative 2 because it gives each person an equal chance to survive. Such a choice reveals aversion to ex-ante inequality. Suppose now, Alternative 2 is compared to a third Alternative 3 in which there is a 50% chance that Person 1 and Person 2 both live and a 50% chance that Person 1 and Person 2 both die. A preference for Alternative 3 reveals aversion to ex-post inequality, i.e. a preference for an equal distribution of realized outcomes.

2 In another scenario, with only one location and a group of ten rescuers, subjects preferred to spread a 10% fatality rate over ten rescuers rather than have one rescuer die for certain. This choice reveals that aversion to ex-ante inequality is stronger than aversion to social risk.

3 For income, they found the opposite pattern, consistent with Keller and Sarin’s (1988) results.

4 Abásolo and Tsuchiya (2020) call Option A the « Inequality scenario» and Option B the « Risk scenario». Choices between these two options are common in the literature. For example, Keller and Sarin (1988) show that individuals tend to prefer the option involving social risk but not ex-post inequality over the option involving ex-post inequality but not social risk. In terms of risk apportionment, such preference for social risk over ex-post inequality is classified as correlation seeking, whereas a preference for ex-post inequality over social risk is classified as correlation averse.

5 With a zero-mean risk, $({t}_{2}-{t}_{1})/2$ is the equalizing transfer in expected value terms.

6 Because the transfer tasks under certainty involve no risk and the transfer tasks under risk involve risk for group 2 only, we did not include the measures of risk aversion for group 1 and cross-temperance in the analysis.

7 The resulting classification gives 62.33 percent of individuals with a majority of risk-averse choices, and 27.4 percent with a majority of risk seeking choices. The remaining 10.27 percent of participants made three choices compatible with risk aversion and three choices compatible with risk seeking. We classified those participants as mixed.

8 If risk aversion is measured for group 2 only, the association between risk aversion and prudence is only marginally significant (Chi-square test, p = 0.09).

9 For the large losses task, the difference in variances was not significant at p-value = 0.66.
