# Supplementary material for: An experimental investigation of social risk preferences for health
Source: Theory Decis. 2023 Apr 27:1–25. Online ahead of print. doi: 10.1007/s11238-023-09928-w (PMC10133917; doi:10.1007/s11238-023-09928-w)
Supplement: Supplementary file 1 — Supplementary file1 (PDF 508 kb) [file 11238_2023_9928_MOESM1_ESM.pdf]

General instructions

Welcome to this experiment. During the experiment:

• please don't talk

• please turn off your cell phone

• please raise your hand if anything is unclear, to be helped in private.

In this experiment, you are asked to make 32 choices. The choices all concern two options, labeled Option A and Option B. The options yield outcomes regarding the life expectancy of persons subdivided in two groups, for convenience labelled Group 1 and Group 2. You can think of these groups being the same. That is, both groups have the same size (that is, an equal number of persons in both groups), and persons in both groups are all similar regarding age, gender, health status etc.

The questions concern allocating reductions in life expectancy across Group 1 and Group 2 in two different formats. We will explain the two formats based on simple examples below. These examples are not choices that you will encounter during the experiment, but simply help you understand the experiment and the two formats.

Examples Format 1 (Instructions sheets U and M)

Below, you see an example of a choice in format 1.

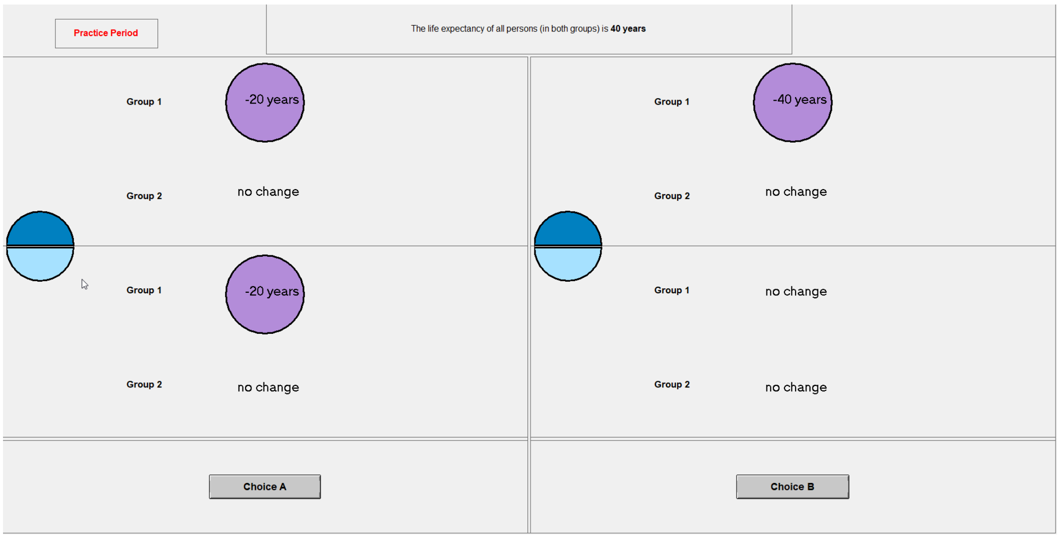

As you can see, the top of the screen shows the period (that is, the choice number) and the amount of time left to make a choice. If the time runs out, you are simply asked to make a choice; you will only proceed to the next choice if you made a choice. Below this, you see the endowment of life expectancy for all persons in the groups; in this case 40 years for all persons in both groups. Below this you see the two options, with Option A on the left side of the screen and Option B on the right side. As you can see, in the example, you see a blue circle with a dark part on top and a light part at the bottom. You can think of this circle as a coin, with a dark blue color on one side, and a light blue color on the other side. If one flips the coin, the probability that the coin lands with the dark side up is the same as the probability that the coin lands with the light side up, both equal to 50%. Hence, the coin indicates (i) that there are two possible outcomes of the flip: dark or light, and (ii) that each state has an equal probability of occurring (that is, 50% —50 out of 100— chance for both). The options A and B yield outcomes for both groups of persons, depending on whether the flip yields dark blue or light blue. The purple circles simply indicate the outcomes in life expectancy. Hence:

- If the flip is dark blue, Option A yields a 20 years reduction in life expectancy for persons in Group 1, and no reduction for Group 2. If light blue comes up, Option A yields a reduction in life expectancy for Group 1 of 20 life years, and no reduction for Group 2. You can think of Option A as Group 1 being affected by some medical condition which for sure will diminish the lifetime of the persons in Group 1 by 20 years.
- Option B yields a 40 years reduction in life expectancy for persons in Group 1, and no reduction for Group 2 if the flip is dark blue. If light blue comes up, Option B yields no reduction in life expectancy, neither for Group 1 nor for Group 2. You can think of Option B

as Group 1 being affected by some medical condition which is more risky than the medical condition of Option A: it will either diminish the lifetime of the persons in Group 1 by 40 years (in case no cure is found), or it will not affect their lifetime at all (in case a cure is found).

Another example choice is given below.

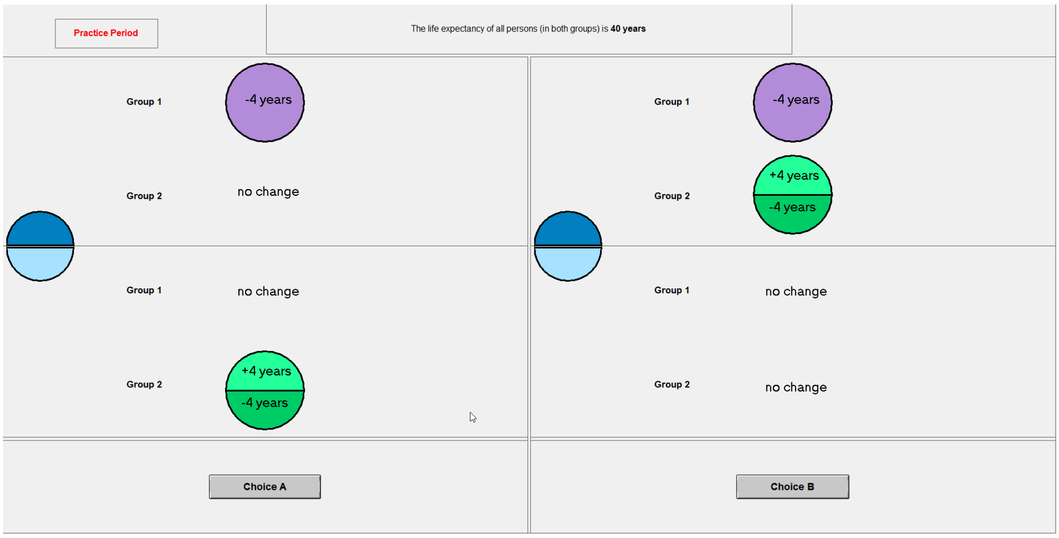

This example is very similar to the previous one, except that the outcomes of the options now also contain a green circle. Like the blue circle, the green circle represents a coin with a light green color on one side and a dark green color on the other side. In particular, in the example, the light green side of the coin contains "+4 life years", while the dark green side of the coin contains "– 4 life years". This indicates the fact that 4 years is added to life expectancy if the green coin lands with the light side up after it is flipped, while 4 years is subtracted from life expectancy if the coin lands with the dark side up. As with the blue coin, the probability that the green coin lands with the dark side up after a flip is the same as the probability that the coin lands with the light side up, that is, both probabilities are equal to 50%.
Hence:

- Option A yields a 4 years reduction in life expectancy for the persons in Group 1, and no reduction for Group 2 if the flip is dark blue. If light blue comes up on the coin, Option A yields no reduction in life expectancy for Group 1, and either 4 years increase (if light green comes up) or a 4 years reduction for Group 2 (if dark green comes up).
- If the flip is dark blue, Option B yields a 4 years reduction in life expectancy for the persons in Group 1, and either a 4 year increase (if light green comes up) or a 4 year reduction for Group 2 (if dark green comes up). If light blue comes up, Option B yields no reduction in life expectancy, neither for Group 1 nor for Group 2.

- You can think of this as the choice where you would prefer to have the green circle: in case there is no change for persons in Group 1 (Option A) or in case there is a 4 year reduction in life years for persons in Group 1 (Option B). In other words, would you prefer a situation where either Group 1 loses 4 life years due to some medical condition or Group 2 faces a health risk that may increase or decrease their lifetime by 4 years (for example, some risky medicine is developed) (A), or a situation where these two events occur simultaneously or both occur not at all (B)?

The choices in format 1 are very similar to the example choices depicted above. Please note that there is no right or wrong answer when choosing between Option A or Option B: the choices you make in the experiment merely reflect your own preference.

Examples Format 2 (Instructions sheet I)

Below, you see an example of a choice in format 2.

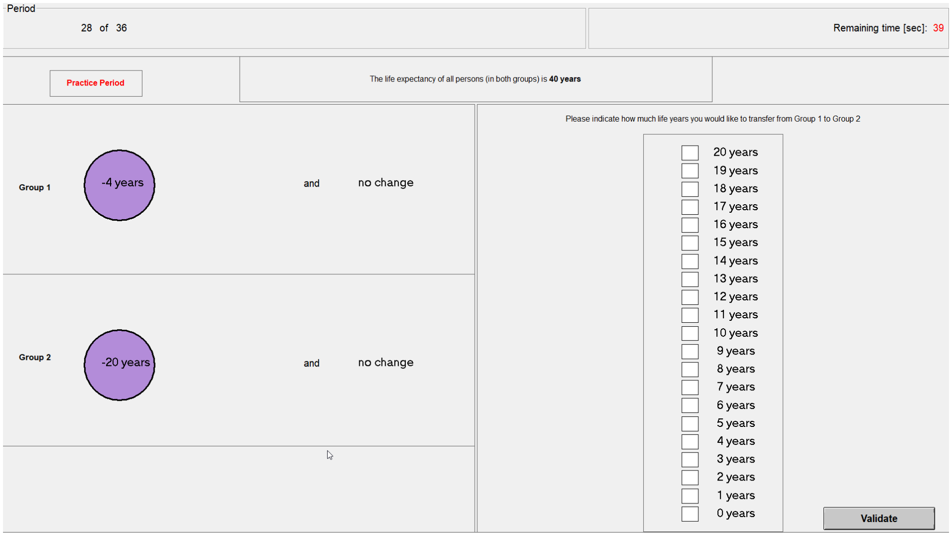

As with the choices in format 1, the top of the screen shows the period (that is, the choice number), the amount of time left to make a choice (which is not binding) and the life expectancy for all persons in both groups, in this case 40 years. Below that, on the left side of the screen, you see changes in the life expectancies for different groups, in this case -4 life years for persons in Group 1, and -20 life years for persons in Group 2. Thus, persons in Group 1 have a life expectancy of 36 years (endowment minus 4), while persons in Group 2 have a life expectancy of 20 years (endowment minus 20). Note that these do not depend on the flip of a coin, that is, these changes are not risky. You can think of this as different life expectancies for the two groups, for example due to genetic variation between these groups.

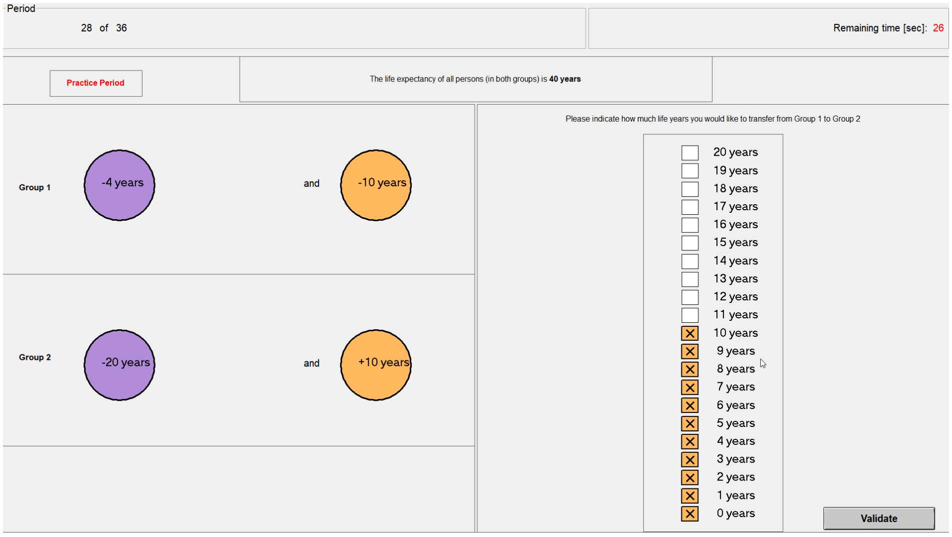

On the right side of the screen, you are asked to indicate how many life years you would like to transfer from Group 1 to Group 2 by selecting one of the checkboxes. Thus, for example, if you select the option “10 years”, 10 years in life expectancy will be subtracted form Group 1, and 10 years will be added to the life expectancy of Group 2. The final allocation of expected life years will thus be +26 life years for persons in Group 1, and +30 life years for Group 2. After selecting the amount of life years you would like to transfer, the left side of the screen will be adjusted based on your choices, that is, an orange circle will appear that shows your adjustment of life years. If you do not want to change the life expectancy of any group, you simply select the checkbox “0 years”. You can think of the orange circles as the amount/quality of medical treatment taken away from one group and provided to the other group. Because of budget constraints, medical treatment provided to one group always comes at the expense of medical treatment given to the other group.

If you are satisfied with your choice you can press “Validate” to continue to the next choice.

Another example choice is given below.

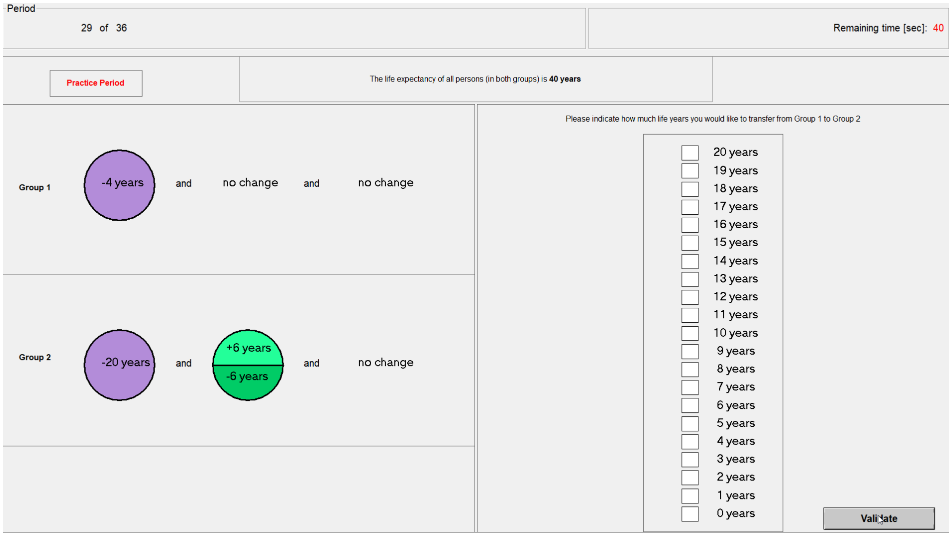

As you can see, the example is very similar to the previous one, except that the outcomes of the options now also contain a green circle. Like the choices in format 1, the green circle represents a coin with a dark green color on one side and a light green color on the other side. The probability that the green coin lands with the dark side up after a flip is the same as the probability that the coin lands with the light side up, that is, both probabilities are equal to 50%. Thus, persons in Group 2 have a life expectancy of 20 years (endowment -20) and either 6 years are added *or* subtracted from their life expectancy with equal (50%) probability. Persons in Group 1 simply have a life expectancy of 36 years.

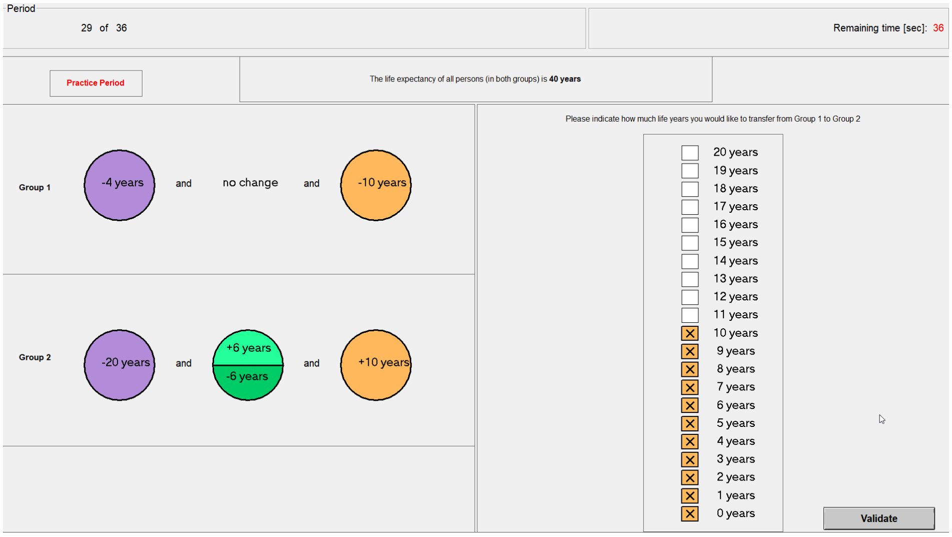

On the right side of the screen, you are again simply asked to indicate how many life years you would like to transfer from Group 1 to Group 2 by selecting one of the checkboxes. After selecting the amount of life years you would like to transfer, the left side of the screen will again be adjusted based on your choices, that is, an orange circle will appear that shows your adjustment of life years. If you do not want to change the life expectancy of both groups, you again select the checkbox “0 years”. If you are satisfied with your choice you can press “Validate” to continue to the next choice.

You can think of the green circles as some additional health risk that is only faced by persons in Group 2. For example, the people in this group may have a more variable life expectancy due to larger genetic variation. The orange circles again reflect the amount of medical treatment transferred from Group 1 to Group 2. Because of budget constraints, medical treatment provided to one group always comes at the expense of medical treatment given to the other group.
The choices in format 2 are very similar to the example choices depicted above. Again, note that there is no right or wrong answer: the choices you make in the experiment merely reflect your own preference.

This ends the instructions. Please raise your hand if you need further explanation from the experimenter. If there are no questions, the experiment will soon start the program. The experiment starts with practice questions. After the practice questions, the experiment will start.
